# Rational Design of Live-Attenuated Vaccines against Herpes Simplex Viruses

**DOI:** 10.3390/v13081637

**Published:** 2021-08-18

**Authors:** Brent A. Stanfield, Konstantin G. Kousoulas, Agustin Fernandez, Edward Gershburg

**Affiliations:** 1 Department of Pathobiological Sciences, School of Veterinary Medicine, Louisiana State University, Baton Rouge, LA 70803, USA; bstanf5@lsu.edu; 2 Division of Biotechnology and Molecular Medicine, Louisiana State University, Baton Rouge, LA 70803, USA; 3 Rational Vaccines Inc., Woburn, MA 01801, USA; af3@rationalvaccines.com

**Keywords:** herpes simplex virus, herpesviruses, live-attenuated vaccines, vaccines

## Abstract

Diseases caused by human herpes simplex virus types 1 and 2 (HSV-1 and HSV-2) affect millions of people worldwide and range from fatal encephalitis in neonates and herpes keratitis to orofacial and genital herpes, among other manifestations. The viruses can be shed efficiently by asymptomatic carriers, causing increased rates of infection. Viral transmission occurs through direct contact of mucosal surfaces followed by initial replication of the incoming virus in skin tissues. Subsequently, the viruses infect sensory neurons in the trigeminal and lumbosacral dorsal root ganglia, where they are primarily maintained in a transcriptionally repressed state termed “latency”, which persists for the lifetime of the host. HSV DNA has also been detected in other sympathetic ganglia. Periodically, latent viruses can reactivate, causing ulcerative and often painful lesions primarily at the site of primary infection and proximal sites. In the United States, recurrent genital herpes alone accounts for more than a billion dollars in direct medical costs per year, while there are much higher costs associated with the socio-economic aspects of diseased patients, such as loss of productivity due to mental anguish. Currently, there are no effective FDA-approved vaccines for either prophylactic or therapeutic treatment of human herpes simplex infections, while several recent clinical trials have failed to achieve their endpoint goals. Historically, live-attenuated vaccines have successfully combated viral diseases, including polio, influenza, measles, and smallpox. Vaccines aimed to protect against the devastation of smallpox led to the most significant achievement in medical history: the eradication of human disease by vaccination. Recently, novel approaches toward developing safe and effective live-attenuated vaccines have demonstrated high efficacy in various preclinical models of herpetic disease. This next generation of live-attenuated vaccines has been tailored to minimize vaccine-associated side effects and promote effective and long-lasting immune responses. The ultimate goal is to prevent or reduce primary infections (prophylactic vaccines) or reduce the frequency and severity of disease associated with reactivation events (therapeutic vaccines). These vaccines’ “rational” design is based on our current understanding of the immunopathogenesis of herpesviral infections that guide the development of vaccines that generate robust and protective immune responses. This review covers recent advances in the development of herpes simplex vaccines and the current state of ongoing clinical trials in pursuit of an effective vaccine against herpes simplex virus infections and associated diseases.

## 1. Introduction

Human herpes simplex virus type 1 (HSV-1) and type 2 (HSV-2) are highly infectious and successful human pathogens. HSV-1 is estimated to infect 3.7 billion people worldwide, with transmission primarily occurring via the oral-to-oral route [1,2,3]. HSV-2 is estimated to infect half a billion people globally, and in the United States, these numbers are predicted to grow by >600,000 new infections per year until 2050 [1]. Infection with either HSV-1 or HSV-2 initially leads to an acute phase of infection in the host’s mucosal epithelium. Productive replication of HSV-1 or HSV-2 in these tissues results in immunopathogenesis, observed as cold sores, blisters, and genital lesions. HSV-1 and HSV-2 infections are generally identified as oral/ocular (HSV-1) and genital (HSV-2) infections. However, HSV-1 causes an increasing proportion of new infections in genital tissues [4,5,6]. Changing sexual practices and the emergence of mutations affecting viral tropism may increase the prevalence of HSV-1 genital infections. A characteristic of all human alphaherpesviruses is their ability to infect and reside in the long-lived sensory neurons of the host’s peripheral nervous system in a transcriptionally repressed state termed “latency” [7]. Epidemiological models predict that a prophylactic vaccine with a modest 50% efficacy can reduce the number of new infections by 58%, incidence by 60%, and seroprevalence by 21%, reducing the yearly rate of infection by 350,000 new cases per year by 2050 [8]. On a population scale, therapeutic vaccination is predicted to reduce new infections by 12%, incidence by 13%, and seroprevalence by 4%, and the number of new infections by 76,000 per year by 2050 [8]. Therapeutic vaccination to reduce or eliminate oral and genital herpes recurrence has long been a highly needed but elusive goal in herpes simplex vaccine development.

Recent advances in our understanding of effective anti-herpes immune responses have led to the development of many novel vaccine approaches [9]. Several academic laboratories and commercial entities are currently working toward developing a safe and effective herpes simplex vaccine in preclinical animal models (Table 1) and human trials (Table 2). In this section, we discuss recent developments in the preclinical pursuit of a safe and effective herpes simplex vaccine and review promising subunit/peptide, vectored/DNA/RNA, and live-attenuated vaccine technologies.

### 1.1. Subunit/Peptide Vaccines

Subunit/peptide vaccines are desirable for vaccine development because they are reasonably stable, safe, and potentially effective. However, subunit-based vaccines against HSV-1 or HSV-2 have fallen short of meeting clinical endpoint criteria. Targeting the major entry mediators of the virus (gB/gD), which are major antigenic determinants, is a primary focus of subunit vaccine development as these immunogens stimulate highly effective neutralizing antibodies [22,23]. Additionally, gE has been utilized in subunit vaccines to target cell-to-cell spread and immune evasion to increase multivalent subunit vaccine efficacy [24,25]. Recently, the field of herpes vaccinology has expanded its focus by using subunit vaccines to include multiple herpes antigens and peptides. Khan et al. demonstrated the efficacy of vaccinating humanized rabbits with HSV-1-derived peptides identified in asymptomatic HSV-1^+^ individuals, followed by chemotactically pulling immune cells into the cornea. This immunization approach protected animals from an ocular challenge [13] (Table 1). The limited antigenic breadth of CD8^+^ T cell peptide epitopes (UL44 aa400–408, UL9 aa196–204, and UL25 aa572–580) reduced vaccine efficacy [13]. However, this “prime/pull” vaccination strategy is an innovative approach to vaccine administration and shows therapeutic efficacy in the most stringent animal models of the HSV-2 disease [26].

Antigenic breadth is of utmost importance when considering effective strategies for herpes vaccines. Previous estimates concluded that herpes simplex encodes 80 open reading frames (ORFs). More recently, with advances in multiomic technologies, it was discovered that HSV-1 encodes 284 ORFs, including 46 novel large ORFs, the functions of which are yet to be elucidated [27]. Considerable effort has been devoted to enhancing vaccine efficacy by increasing the number of antigens included in formulations. For instance, a bivalent HSV-2 subunit (gD2 and gB2) vaccine delivered by intranasal (IN) immunization elicited increased neutralizing antibody titers compared to a monovalent gD2 vaccine delivered IN. However, it was less effective than gD2-alone delivered intramuscularly. The bivalent gD2/gB2 vaccine reduced acute and recurrent disease scores and latent viral load compared to placebo. Therapeutically, IN vaccination reduced recurrent lesion sores, days with the disease, animals shedding virus, and virus-positive vaginal swabs [14].

Significant morbidity and mortality are associated with neonatal herpes simplex infections. Children that survive neonatal HSV infection may develop lifelong developmental and behavioral disorders. Vaccination of pregnant mice with a trivalent herpes simplex vaccine (gD2, gE2, and gC2) protected mice and offspring against lethal challenge with virulent HSV-1 and HSV-2 strains. Importantly, neonatal mice were protected from developing long-term behavioral morbidity [15]. These results suggest that an effective vaccination strategy can successfully combat neonatal infections.

### 1.2. Vectored/DNA/RNA Vaccines

Messenger RNA (mRNA)-based vaccines are promising technologies, which have been rapidly and successfully deployed against SARS-CoV-2, the causative agent of COVID-19. Moderna and Pfizer (in collaboration with BioNTech) have successfully launched two synthetic mRNA vaccines expressing the viral spike (S) glycoprotein. Both vaccines have been extensively used to vaccinate many people and proven to be safe and efficacious against SARS-CoV-2 infections, even conferring significant protection against variants that increase the rate of transmission [28,29]. With the demonstrated efficacy of mRNA vaccines under emergency use authorization, many novel mRNA-based vaccines are being pursued for other infectious disease pathogens, including herpesviruses. Specifically, Awashi et al. [11] demonstrated a trivalent, nucleoside-modified mRNA vaccine’s efficacy in preventing clinical and subclinical genital HSV-2 disease in both mouse and guinea pig models of genital HSV-2 infection. Vaccination prevented the formation of genital lesions following guinea pigs and mice challenged with HSV-2. Additionally, two doses at 10 μg of the trivalent mRNA vaccine outperformed the three doses at 5 μg each of the trivalent subunit vaccine. The mRNA vaccination scheme stimulated superior systemic and vaginal HSV-2 specific IgG, neutralizing antibodies, and gD2 specific antibodies. This mRNA vaccine demonstrated superior immunogenicity, as evidenced by the stimulation of long-lived CD4^+^ T cells, T follicular helper cells, and germinal center B cell responses. This trivalent mRNA vaccine is a promising candidate for future clinical trials in humans [11].

DNA has been proposed to be a valuable tool in the development of an effective HSV vaccine. It can be rapidly synthesized, purified, and is more stable than mRNA. Bagley et al. demonstrated that a DNA vaccine expressing a pool of HSV-2 glycoproteins (gB2, gC2, gD2, gE2, gH2, gL2, and gI2) adjuvanted with IL-12 outperformed the gD2 subunit vaccine but was not as effective in reducing virus shedding compared with the HSV-2 0ΔNLS live-attenuated vaccine. Thus, despite including seven prominent HSV-2 antigens, this DNA vaccine was not as effective as the live-attenuated vaccine. This work highlights the importance of antigenic breadth in vaccine efficacy against genital HSV-2 infection, which can only be reproduced effectively through an active infection with a live-attenuated virus [10]. To this end, live-attenuated vaccines have repeatedly demonstrated superior efficacy.

Vectored vaccines are becoming increasingly prevalent. Many of the vaccines deployed to combat the SARS-CoV-2 pandemic are replication-defective adenovirus vector vaccines expressing the SARS-CoV-2 spike (S) glycoprotein. These vaccines have demonstrated efficacy in preventing disease. Recently, safety concerns have stemmed from rare cardiac thrombotic and cardiomyopathy incidences of unexplained origin, which may be due to the vehicle carrier, and potential inflammatory properties of mRNA and the adenovirus vectors used to express the S glycoprotein. Vaccine stability is always a primary concern. Synthetic mRNA technology utilizes modified RNA nucleotides that may produce unwanted side effects and require very low temperatures to sustain vaccine stability. Adenovirus-based vaccines are much more stable than mRNA vaccines and do not require ultra-low temperatures for stability. Vaccinia (poxvirus)-based vaccines are extensively used due to their demonstrated efficacy in combating the smallpox epidemic. More recently, vaccinia vectors have been developed with improved safety profiles to be avirulent and induce high levels of transgene expression in the modified vaccinia virus Ankara (MVA) background. The MVA vector technology has demonstrated efficacy against HIV, influenza, parainfluenza, measles, flavivirus, and even malaria [30]. Atukorale et al. developed a vaccinia virus vectored HSV-2 vaccine expressing gD2 [12]. However, the transgene was lost following the vaccine’s serial passage, pointing to potential vaccine stability issues. The authors also demonstrated that the transgene insertion site could dictate vector stability with a prolonged serial passage in cells, indicating that vaccinia vectors can be a viable platform for sound engineering. 

### 1.3. Live-Attenuated Vaccines

Live-attenuated vaccines have been the most effective vaccines to combat human and animal viral infections in medical history. The repertoire of these successes includes the eradication of smallpox, poliomyelitis, measles, mumps, rubella, rotavirus, and others (reviewed in: [31]). A live-attenuated varicella-zoster virus (VZV) (human herpesvirus 3 (HHV-3) or chickenpox virus) vaccine is widely used worldwide and shown to be highly efficacious in controlling viral reactivation. Live varicella vaccine is generally safe and well-tolerated [32]. The success of the alphaherpesvirus VZV live-attenuated vaccines provides a primary example suggesting that a similar approach may be efficacious in combating herpes simplex infections which, like VZV, establish latency in neurons. In addition, the only FDA-approved oncolytic virotherapy on the market is a live-attenuated herpes simplex virus (TVEC or Imlygic) approved for the treatment of human melanoma. This virus was designed as an oncolytic and immunotherapeutic virus that augments anti-tumor immunological responses. Imlygic is a replication-competent HSV-1 mutant strain, with the deletion of both γ34.5 and ICP47 genes. These deletions limit virus replication in cancer cells and eliminate the inhibition of antigen presentation by the ICP47 gene. Additionally, the virus expresses human GM-CSF, which stimulates the recruitment of antigen presenting cells providing enhanced immunogenicity [33].

The generation of a safe and effective herpes simplex vaccine must focus on preparing attenuated viruses that can generate robust immune responses. HSV-1 and HSV-2 share ~83% of nucleotide identity, and cross-protective immunity may be achieved due to the extensive repertoire of cross-protective antigens [34]. To this end, novel live-attenuated vaccine strategies are being implemented to tame the virus in vivo. The replication-competent HSV-1 vectors (HSV-GS3 and HSV-GS7) demonstrated prophylactic efficacy in a mouse model of dermal HSV-1 infection. These vectors are controlled by placing one or two essential genes under the stringent control of a gene switch coactivated by heat and antiprogestin. In the absence of these activating factors, the controlled HSV-1 vectors do not replicate. In this study, the unactivated HSV-1 vectors offer equivalent protection to chemically inactivated vaccines. However, the activation of these controlled HSV-1 vectors increases vaccine efficacy over inactivated vaccines [16].

Recently the use of live-attenuated HSV-1 vaccines has demonstrated robust protection against ocular HSV-1 disease. More specifically, the HSV-1 0ΔNLS lacking a nuclear localization signal of the viral ubiquitin ligase ICP0, and the non-neurotrophic HSV-1 vaccine vector VC-2 with deletions in the amino terminus of both the gK and UL20 genes demonstrated effective protection against ocular HSV-1 challenge. Mice vaccinated with HSV-1 0ΔNLS showed superior protection against early viral replication, neuroinvasion, latency, and mortality than gD-2-vaccinated or naive mice following ocular challenge with a neurovirulent clinical isolate of HSV-1. Moreover, 0ΔNLS-vaccinated mice exhibited protection against ocular immunopathology and maintained corneal mechanosensory function. Vaccinated mice also showed suppressed T cell activation in the draining lymph nodes following the challenge. Vaccine efficacy correlated with serum neutralizing antibody titers. Humoral immunity was identified as a significant correlate of protection against corneal neovascularization, HSV-1 shedding, and latency through passive immunization [35,36].

Interestingly, vaccination with the VC-2 vaccine protected mice from developing any appreciable ocular pathology, while vaccination with the attenuated parental HSV-1 (F) strain only offered partial protection. The corneas of VC-2 immunized mice demonstrated a significantly increased infiltration of T cells and limited infiltration of Iba1^+^ macrophages compared to unvaccinated or parental HSV-1 (F) strain vaccinated groups. Animals vaccinated with VC-2 produced higher neutralizing antibody titers than the parental HSV-1 (F) strain post-challenge. Vaccination with VC-2 significantly increased the CD4 T central memory (TCM) subsets and CD8 T effector memory (TEM) T cell subsets in the draining lymph nodes following the ocular HSV-1 (McKrae) challenge, than unvaccinated mice or mice vaccinated with parental HSV-1 (F) strain, indicating that VC-2′s immunogenicity is superior to wild-type HSV-1 vaccination [21].

Neurotropism is the main hallmark of alphaherpesviruses and a major challenge in designing live-attenuated viruses that ideally should not establish latency in neurons. Recent attempts to inhibit virus entry into neuronal axons have yielded several novel live-attenuated HSV-1 vectors. The HSV-1 R2 live-attenuated vaccine has five missense mutations in the UL37 gene. UL37 has been shown to play a conserved role in alphaherpesvirus neurotropism by facilitating retrograde virion transport upon infection of neuronal axons [37]. The mutations in the R2 live-attenuated vaccine disrupt neuronal retrograde transport rendering the virus unable to establish latent infection in the nucleus of the neuronal cell body. In the guinea pig model of genital HSV-2 disease, intradermal (ID) vaccination with the R2 vaccine demonstrated superior performance to intramuscular (IM) vaccination with the gD2 monovalent subunit vaccine. Similarly, ID vaccination with R2 induced higher neutralizing antibody titers than IM vaccination with the gD2 subunit vaccine alone [20]. In comparison, the non-neuroinvasive HSV-1 VC2 vaccine demonstrated superior protection to the gD2 subunit vaccine while generating long-lasting efficacy up to 6 months post-vaccination in a guinea pig model of genital HSV-2 infection [19,38].

## 2. Rational Design of the VC-2 Vaccine

### 2.1. The Structure and Function of Glycoprotein K and the Membrane Protein UL20

Glycoprotein K (gK) is a highly hydrophobic glycoprotein having four transmembrane domains placing both amino and carboxy termini extracellularly. Glycoprotein K (gK) is required for efficient virus envelopment and functions as a heterodimer with the membrane protein UL20 [39,40]. The majority of mutations that cause enhanced virus-induced cell fusion are found within gK mediated primarily by interactions between gK and the amino terminus of gB [41,42,43,44]. Together gK and UL20 are highly conserved among neurotrophic alphaherpesviruses indicating highly conserved functions within this virus family [45,46,47]. Additionally, the amino terminus of gK is required for the interaction between gB and the cellular protein Akt. Upon binding to gB, Akt is phosphorylated and induces calcium release from the cell [48].

### 2.2. Herpes Simplex Virus Mechanism of Entry—Rational Design of the VC2 Live-Attenuated Vaccine

HSV is known to enter into cells by two mechanisms: fusion (between the viral envelope and the cellular plasma membrane) and endocytosis. Fusion requires the formation of a multiprotein complex including gB, gD, gH/gL, and their cellular receptors. This complex is known as the “core fusion machinery” and is essential for HSV entry into cells [49,50,51,52]. Membrane fusion is initiated upon gD binding to one of its cellular receptors (HVEM, Nectin-1, Nectin-2), which induces a conformational change in gD and activates gH/gL to act upon gB changing from its prefusion conformation to its postfusion conformation. These conformational changes induce fusion between the viral envelope and the host cell membrane by releasing the viral contents into the cell’s cytoplasm [53].

In some cases, HSV will enter by endocytosis, resulting in a double-membraned virion in the cytoplasm. Escape from endocytic vesicles is pH-dependent; however, release from the endocytic vesicle ultimately requires fusion of the viral envelope with the membrane of the endocytic vesicle [54]. It has been proposed that the switch between endocytosis and direct fusion occurs upon gB binding to the cell surface receptor PILRα [55]. Specifically, this mechanistic switch is known to occur via interactions between gB and gK. HSV-1 lacking the amino terminus of gK (AA31-68) can no longer enter into PILRα cells, indicating gK mediates this phenotype [56]. However, entry into the axons of sensory neurons is strictly dependent on the direct fusion between the viral envelope and axonal cellular membranes [57]. Disruption of the ability of gK to induce gB-mediated membrane fusion restricts HSV-1 to replication in epithelial cells allowing for the presentation of all viral antigens. At the same time, the virus cannot establish latency in neurons. Interestingly, viral entry through endocytosis by the HSV-1 (VC-2) vaccine strain upregulated type I interferon responses and the induction of certain chemokines such as CXCL4 and TNF, which may result in the observed increased immunogenicity of VC2 compared to its parental wild-type virus HSV-1(F) (Clark and Kousoulas, unpublished).

## 3. Rational Design of the HSV-2 0∆NLS Vaccine

### 3.1. The HSV ICP0 Protein

ICP0 is a ~775 amino acid polyfunctional protein, an E3 ubiquitin ligase bearing a RING-finger domain. The mature length of the ICP0 polypeptide may vary, depending on the HSV isolate. ICP0 is known to play a key role in activating HSV-1 gene expression, the disruption of ND10 structures, degrade cellular proteins via polyubiquitination, and evasion of the host cell’s antiviral defenses [58]. Initially identified as a viral factor for viral replication in vitro, the ICP0-null virus demonstrated a significant growth defect, especially at low multiplicities of infection [59,60,61,62,63]. Subsequent in vivo experiments demonstrated the requirements of ICP0 for productive replication and reactivation from latent infection [64,65,66,67]. ICP0 is capable of transactivating promoters involved in all three phases of lytic transcriptional activation (α, β, and γ genes) [68,69,70,71]. Recent publications have identified ICP0 as a critical mediator of host cell chromatin architecture via the targeted degradation of the cellular epigenetic machinery. Specifically, ICP0 targets Schlafen 5 (SLFN5) for proteasomal degradation via ubiquitination of SLFN5. Infection with virus lacking ICP0 (HSV-1ΔICP0) SLFN5 binds to viral DNA to repress transcription via limiting RNA polymerase II access to immediate-early viral promoters [72]. Similarly, TRIM22 has been identified as an intrinsic host cell factor limiting HSV replication via the viral genome’s heterochromatinization. ICP0 has been shown to disrupt this intrinsic defense in a mechanism independent of ICP0’s ubiquitin ligase activity [73]. Additionally, ICP0 interacts with promyelocytic leukemia protein (PML) and facilitate its SUMO-independent degradation as a viral countermeasure to circumvent PML-mediated antiviral restrictions [74].

### 3.2. The Role of ICP0 Protein in Immune Evasion

Host cell recognition of cytosolic viral DNA is crucial for early antiviral defenses. Several cytosolic DNA sensors serve as pattern recognition receptors (PRRs) in the host cell defense against HSV infection. Interferon Gamma Inducible Protein 16 (IFI16) binds double-stranded DNA and is a member of the PYHIN family of proteins. IFI16 is primarily localized to the nucleus but shuttles between the nucleus and cytoplasm via acetylation by the histone acetyltransferase p300. Upon infection with HSV-1, IFI16 recognizes viral DNA in the nucleus and is rapidly acetylated for distribution to the cytoplasm, where it activates interferon production to restrict viral replication [75,76]. IFI16 is targeted by ICP0 for ubiquitination and degradation by the cellular proteasome [77,78,79]. Similarly, ICP0 has been shown to target IRF7 to limit antiviral interferon production [80].

The nuclear factor kappa-light-chain-enhancer of activated B cells (NF-κB) plays an essential role in regulating inflammation. ICP0 has been shown to suppress TNF-α mediated NF-κB activation via interaction with the p65 and p50 NF-κB subunits. ICP0 inhibits p65 nuclear translocation following TNF-α stimulation. ICP0 also actively targets the p50 NF-κB subunit for proteolytic degradation due to its E3 ubiquitin ligase activity. This ability to inhibit NF-kB activity is dependent on the RING-finger domain (RFD) of ICP0, and the RFD alone was sufficient to inhibit NF-κB activity in reporter assays [81].

HSV ICP0 is reported to alter the adaptive immune response to HSV infection significantly. A proposed mechanism for this property is ICP0’s ability to inhibit CD83 expression in HSV-infected dendritic cells. This phenotype is mediated by the ICP0 RFD and is dependent on the proteolytic degradation of CD83 [82]. Also, ICP0 inhibits important components of the antigen presentation pathway in sentinel cells, which are responsible for activating the adaptive arm of the immune system. Virus lacking ICP0 is more immunogenic, increasing the breadth of antigen recognition by antibodies from immunized animals and generating superior protection compared with the gD2 subunit vaccine in mice and guinea pig models of genital HSV-2 disease [83,84,85].

Recently, it was reported that HSV-1 DNA activates the host cell DNA damage response (DDR) kinase pathways. Specifically, in cells infected with ICP0-null HSV-1, components of the host cell DDR facilitated viral replication (ATM and p53) or restricted viral replication (Mre11). However, ICP0 expression ablated these DDR effects, indicating ICP0 plays an important role in the viral evasion of host cell DDR [86]. Apparently, deletion of ICP0 unlocked the immune response to HSV infection, leading to greater antigen presentation and altering the inflammatory response to HSV infection. The polyfunctionality of the ICP0 protein establishes it an ideal target for vaccine and vector attenuation.

## 4. Clinical Trials of Herpes Simplex Vaccines

The only ongoing clinical trial evaluating the safety and efficacy of an HSV-2 vaccine is the Sanofi-Pasteur SP0148 vaccine (Table 2). The primary endpoints of this clinical trial include: (1) Evaluation of the safety profile of different investigational vaccine regimens against HSV-2; (2) evaluation of the relative efficacy of investigational vaccine regimens concerning the frequency of HSV shedding by PCR to detect viral DNA in the genital area (shedding rate) following the two-dose vaccine schedule; (3) determining the proportion of participants free of HSV genital recurrence at 6 months after the two-dose vaccine schedule. The secondary objectives of this study include: (1) Evaluating the impact of each investigational vaccine regimen in terms of the total number of days with genital lesions up to 6 months after the second vaccination, and number of recurrences 60 days after the second vaccination compared to the placebo group; (2) describing the efficacy of each investigational vaccine regimen concerning the frequency of HSV DNA detection in the genital area (shedding rate) 60 days following the first vaccination visit plus 60 days following the second vaccination visit compared to the placebo group; (3) describing the efficacy of the investigational vaccine regimens with respect to HSV DNA detection in the genital area (shedding rate) 60 days after the first vaccination visit compared to the placebo group. 

Despite the historical failure to deliver an FDA-approved successful vaccine strategy by existing vaccine approaches, much knowledge has accumulated from past and ongoing studies regarding immunological features required for successfully confronting herpes simplex infections. Several novel vaccine approaches are in late-stage preclinical development, moving toward phase 1 trials in the coming years (Table 2). These approaches will likely demonstrate varied efficacy in clinical trials; although, they have demonstrated high efficacy in preclinical animal models. It is important to note that there were reports that an exacerbated CD8+T cell response through repeated reactivation may increase the rate of reactivation [87]. Therefore, an effective vaccine approach is required to protect without exacerbating the reactivation of the latent virus, suggesting that both inflammatory and immune-regulatory pathways and cellular milieus must be considered.

## 5. Conclusions

Live-attenuated, highly safe viruses generate a robust immune response, such as HSV-1 (VC2) and HSV-2 0∆NLS, provide a promising approach as both prophylactic and therapeutic vaccines against HSV-1 and HSV-2 infections. Forthcoming phase I and II clinical trials are needed to provide necessary human data showing that these vaccines are well-tolerated while generating effective and broadly therapeutic immune responses. Mutating highly conserved genes within the herpes virus, used to circumvent the host’s innate and adaptive immune responses, represents a novel strategy in the rational development of live-attenuated herpes vaccines. The HSV-2 0∆NLS and VC2 vaccines represent two examples of this rational design approach. Importantly, these viruses can also be efficiently used as vectors for producing vaccines against other human pathogens due to their ability to express multiple genes as insertions into their genomes without appreciably affecting their viral infectivity.

## Figures and Tables

**Table 1:** Current herpes simplex vaccines under preclinical development.

Type of Vaccine	Description	Adjuvant	Type of Study	Animal Model	Route of Challenge	Results	Year	Refs.
Vectored/DNA/RNA	Polyvalent HSV-2 glycoprotein DNA vaccine (gB2, gC2, gD2, gE2, gH2, gL2, and gI2)	DNA encoding IL-12	P	Mouse (Balb/c)	Genital HSV-2	DNA vaccines targeting optimal combinations of surface glycoproteins provide better protection than gD alone and similar survival benefits and disease symptom reductions compared with a potent live-attenuated HSV-2 0ΔNLS vaccine. However, mice vaccinated with HSV-2 0ΔNLS clear the virus much faster.	2017	[10]
Vectored/DNA/RNA	Nucleoside-modified mRNA encoding HSV-2 gC2, gD2, and gE2	Lipid nanoparticle (LNP)	P	Mouse (Balb/c) and guinea pig (Hartley)	Genital HSV-2	The trivalent mRNA vaccine outperformed trivalent subunit-based vaccines, reducing latent viral load, shedding infectious virus, and PCR positive vaginal swabs.	2019	[11]
Vectored/DNA/RNA	Modified vaccinia virus Ankara (MVA) expressing HSV-2 gD2	NA	Vector Stability	NA	NA	Serial passage of recombinant vaccinia vectors led to the loss of transgene expression	2020	[12]
Subunit	Asymptomatic CD8+ T cell peptide epitopes (UL44 aa400–408, UL9 aa196–204, and UL25 aa572–580)	CpG (Prime) followed by AAV8 vectored CXCL10 (Pull)	P	HLA transgenic rabbits	Ocular HSV-1	Prime/pull was effective at drawing HSV-1-specific CD8+ T cells to the cornea and trigeminal ganglia, reducing disease.	2018	[13]
Subunit	Bivalent HSV-2 Subunit (gD2 and gB2)	Nanoemulsion adjuvant NE01	P/T	Guinea pig (Hartley)	Genital HSV-2	Intranasal (IN) vaccination significantly reduced acute and recurrent disease scores and latent viral load compared to a placebo. Therapeutically, IN vaccination reduced recurrent lesion sores, days with the disease, animals shedding virus, and virus-positive vaginal swabs.	2019	[14]
Subunit	Trivalent HSV-2 subunit vaccine (gC2, gD2, and gE2)	CpG (5′-TCCATGACGTTCCTGACGTT-3’)/Alum	P	Neonatal Mouse (C57BL/6)	Intranasal (HSV-1/HSV-2)	Maternal vaccination protected offspring against neonatal disseminated disease and mortality from HSV-1 and HSV-2.	2020	[15]
Live-Attenuated	Replication-Competent Controlled HSV-1 Vectors (HSV-GS3 and HSV-GS7)	NA	P	Mouse (Swiss Webster)	Rear Footpad HSV-1	Inactivated HSV-1 vectors offered equivalent protection to inactivated vaccines. Activation of these controlled vaccines increased vaccine efficacy over inactivated vaccines.	2018	[16]
Live-Attenuated	Replication-defective HSV-2 dl5-29 (Lacking UL5 and UL29)	NA	P	Mouse (C57BL/6) and Neonatal Mouse (C57BL/6)	Adult Ocular (Corneal HSV-1 infection), Neonatal Mouse (Intranasal HSV-1 Infection)	Maternal vaccination led to the transfer of HSV-specific antibodies into neonatal circulation that protected against neonatal neurological disease and death.	2019	[17]
Live-Attenuated	HSV-1 0ΔNLS	NA	P	Mouse (C57BL/6)	Ocular HSV-1	Sterile immunity to ocular HSV-1 challenge with reduced infection of the nervous system. Vaccination preserved cornea free of pathology and complete preservation of visual acuity.	2019	[18]
Live-Attenuated	The non-neuroinvasive VC2 HSV-1 vaccine (Deletion of gK aa31-68 and UL20 aa4-22)	NA	P	Guinea pig (Hartley)	Genital HSV-2	The live-attenuated VC2 vaccine outperformed the gD2 subunit vaccine in the durability of vaccine-induced protection 6 months post-vaccination.	2019	[19]
Live-Attenuated	R2 non-neuroinvasive HSV-1 vaccine (HSV1-GS6264, 5 missense mutations in UL37)	NA	P	Guinea pig (Hartley)	Genital HSV-2	The live-attenuated prophylactic HSV vaccine, R2, was effective in the guinea pig model of genital HSV-2, especially when administered by the ID route.	2020	[20]
Live-Attenuated		NA	P	Mouse (Balb/c)	Ocular HSV-1	VC2 vaccination in mice produced superior protection and morbidity control compared to its parental strain HSV-1 (F).	2020	[21]

Abbreviations: P—Prophylactic, T—Therapeutic.

**Table 2:** Recent/current/pending clinical trials of herpes simplex vaccines.

Sponsor	Intervention	Summary	Status	ClinicalTrials.gov Identifier
Sanofi Pasteur	SP0148 (also known as ACAM 529 or HSV 529), a defective replication HSV-2 with deletions in UL5 and UL29	Estimated enrollment of 381 HSV-2 seropositive patients	Active, not recruiting; Phase 1/2	NCT04222985
Genocea Biosciences	GEN-003 is a subunit vaccine comprising HSV-2 glycoprotein D2 (gD2ΔTMR_340–363_) and infected cell polypeptide 4 (ICP4_383–766_) adjuvanted with proprietary Matrix-M2	Genocea Biosciences, Inc. announced that they entered into a material transfer agreement and exclusive license option with Shionogi & Co., Ltd.	Terminated; Phase 2	NCT03146403
Vical	VCL-HB01 Plasmid-based vaccine encoding two HSV-2 proteins and VCL-HM01 Plasmid-based vaccine encoding one HSV-2 protein, both adjuvanted with Vaxfectin	VCL-HB01 was ineffective in reducing outbreaks in people who were infected with HSV-2	Completed; Phase 2	NCT02837575
Agenus	HerpV polyvalent peptide complex adjuvanted with QS-21	Stopped after Phase 2	Completed; Phase 2	NCT01687595
X-Vax Technology	HSV-2 ΔgD-2	Preparing for a Phase 1 clinical study	Preclinical	NA
UPenn in collaboration with BioNTech	HSV-2 mRNA vaccine coding gC2, gD2, and gE2	Preparing for a Phase 1 clinical study	Preclinical	NA
Rational Vaccines	RVx201 (derivative of HSV-2 0∆NLS)	Preparing for a Phase 1 clinical study	Preclinical	NA
Rational Vaccines	RVx1001 (HSV-1 VC2)	Preparing for a Phase 1 clinical study	Preclinical	NA

## Data Availability

Not applicable.
